# Perinatal Penicillin Exposure Affects Cortical Development and Sensory Processing

**DOI:** 10.3389/fnmol.2021.704219

**Published:** 2021-12-22

**Authors:** James Perna, Ju Lu, Brian Mullen, Taohui Liu, Michelle Tjia, Sydney Weiser, James Ackman, Yi Zuo

**Affiliations:** Department of Molecular, Cell and Developmental Biology, University of California, Santa Cruz, Santa Cruz, CA, United States

**Keywords:** penicillin, somatosensory cortex, inhibitory interneuron, perineuronal net, dendritic spine, microglia

## Abstract

The prevalent use of antibiotics in pregnant women and neonates raises concerns about long-term risks for children’s health, but their effects on the central nervous system is not well understood. We studied the effects of perinatal penicillin exposure (PPE) on brain structure and function in mice with a therapeutically relevant regimen. We used a battery of behavioral tests to evaluate anxiety, working memory, and sensory processing, and immunohistochemistry to quantify changes in parvalbumin-expressing inhibitory interneurons (PV+ INs), perineuronal nets (PNNs), as well as microglia density and morphology. In addition, we performed mesoscale calcium imaging to study neural activity and functional connectivity across cortical regions, and two-photon imaging to monitor dendritic spine and microglial dynamics. We found that adolescent PPE mice have abnormal sensory processing, including impaired texture discrimination and altered prepulse inhibition. Such behavioral changes are associated with increased spontaneous neural activities in various cortical regions, and delayed maturation of PV+ INs in the somatosensory cortex. Furthermore, adolescent PPE mice have elevated elimination of dendritic spines on the apical dendrites of layer 5 pyramidal neurons, as well as increased ramifications and spatial coverage of cortical microglia. Finally, while synaptic defects are transient during adolescence, behavioral abnormalities persist into adulthood. Our study demonstrates that early-life exposure to antibiotics affects cortical development, leaving a lasting effect on brain functions.

## Introduction

Antibiotics have revolutionized medicine and saved countless lives. Over the past few decades, they have been extensively used in pediatric care in developing and developed countries alike (Rogawski et al., 2017; Youngster et al., 2017). In the United States, medical professionals prescribe antibiotics, particularly β-lactam antibiotics such as penicillin, more than any other drug to infants and young children (Hicks et al., 2015; Hales et al., 2018). Furthermore, antibiotics are widely used during pregnancy and as intrapartum prophylaxis, which may indirectly expose the child to antibiotics (Stokholm et al., 2013; Persaud et al., 2015). However, accumulating evidence suggests that early-life antibiotic exposure may have long-lasting adverse effects on the health and wellness of children (Clausen et al., 2016; Ahmadizar et al., 2017), particularly with regards to brain development (Rogers et al., 2016). Early-life antibiotic exposure has been epidemiologically correlated with lower intelligence, reading, and social scores as well as higher behavioral difficulty scores, suggesting that it may be a risk factor for attention deficit (hyperactivity) disorder (ADD/ADHD) as well as depression and anxiety disorders (Slykerman et al., 2017). Penicillin exposure in the second and third trimesters have also been associated with an increased risk of autism spectrum disorder (ASD) (Atladottir et al., 2012). Corroborating these findings, rodents subjected to various antibiotic regimens have been found to exhibit a number of behavioral abnormalities, including decreased sociability (Desbonnet et al., 2014; Leclercq et al., 2017), increased anxiety-like behaviors (Tochitani et al., 2016), and increased visceral hypersensitivity (O’Mahony et al., 2014).

Sensory processing refers broadly to the processes in which the animal receives, interprets, integrates, and responds to sensory information. It develops through early life in an experience-dependent manner (Lickliter, 2011). Abnormal sensory processing has been observed in a number of neurodevelopmental disorders including ADHD (Mangeot et al., 2001; Parush et al., 2007) and ASD (Ben-Sasson et al., 2009; Marco et al., 2011), in which defects are strongly associated with symptoms of depression and anxiety (Reynolds and Lane, 2009; Mazurek et al., 2013; Bitsika et al., 2016). In these diseases, sensory processing defects may manifest as hyper- or hypo-sensitivity to sensory stimuli as well as impaired discrimination abilities (Ghanizadeh, 2011; American Psychiatric Association, 2013). As defects in sensory processing can be quantitatively measured and analyzed in rodent models as well as human patients, they may serve as biomarkers for neurological and neuropsychiatric disorders (Sinclair et al., 2017; Harrison et al., 2019).

Synapses, the sites where neurons communicate with each other, are fundamental units of information processing in the brain (Sudhof and Malenka, 2008). Neural circuits in the mature brain emerge from the pruning of supernumerary synapses, and neural circuit reconfiguration hinges upon the continual formation and elimination of synapses (Holtmaat and Caroni, 2016; Bennett et al., 2018). Abnormal synaptic pruning thus leads to circuit mis-wiring and abnormal neuronal activities, and underlies various neurodevelopmental and neuropsychiatric disorders (Keshavan et al., 1994; Irwin et al., 2000; Thomas et al., 2016). In addition, the synaptic circuit is shaped by microglia, the resident immune cells in the central nervous system (Mosser et al., 2017). Microglia tile the neural parenchyma, exhibit a ramified morphology, and continuously extend and retract their processes to survey their immediate environment (Davalos et al., 2005; Thion and Garel, 2017). Recent studies further show that microglia contribute to the modulation of neuronal activity as well as synaptic dynamics and plasticity (Tremblay et al., 2010; Li et al., 2012). During postnatal brain development, they facilitate synaptic pruning by complement activation and subsequent phagocytosis (Paolicelli et al., 2011; Schafer et al., 2012); in adolescent and mature brains they regulate experience-dependent synaptic plasticity (Tremblay et al., 2010; Rogers et al., 2011; Parkhurst et al., 2013; Sipe et al., 2016). Altered microglial morphology, dynamics, and interactions with neuronal synapses have been found in many neurological and psychiatric diseases (Yirmiya et al., 2015; Tay et al., 2017; Yokokura et al., 2021).

In this study, we investigated how early-life antibiotic exposure affects sensory processing in a mouse model. We subjected mice to a therapeutically relevant perinatal penicillin exposure (PPE) regimen. We found that PPE significantly increased sensorimotor gating and decreased the ability to discriminate between textures. Such behavioral alterations were accompanied by increased spontaneous neuronal activities and delayed maturation of inhibitory neuronal circuits. Furthermore, PPE accelerated synaptic pruning on cortical excitatory neurons and increased the ramification and structural dynamics of microglia therein. Our findings shed light on the potential mechanisms underlying the long-term effects of PPE on the developing brain.

## Materials and Methods

### Experimental Animals

Snap25-GCaMP6s (JAX#025111), *Thy1*-YFP-H (JAX#003782), and Cx3cr1-GFP (JAX#005582) mouse lines were purchased from The Jackson Laboratory and maintained on the C57BL/6J background. Mice were group-housed in the UCSC animal facility, with 12 h light-dark cycle and access to food and water *ad libitum*. Mice of both sexes were used in the study. All animal studies were performed in accordance with protocols approved by the Institutional Animal Care and Use Committee (IACUC) of UCSC.

### Perinatal Penicillin Exposure

We followed a published PPE regimen (Leclercq et al., 2017). Briefly, phenoxymethylpenicillin (penicillin V) was administered to pregnant dams in the drinking water (31 mg/kg bodyweight per day) from embryonic day 16 (E16) to postnatal day 15 (P15).

### Behavioral Tests

Home cages were moved from the colony room to the behavior room at least 30 min prior to any handling or experiment. Animals were handled 5 min per day for 2 days in the behavior room prior to any behavioral test to acclimate them to the experimenter and the environment.

#### Spontaneous Alternation Y-Maze

The Y-maze is an opaque plastic arena composed of three 35 cm × 7 cm arms diverging at 120° angles. During the test, the subject mouse was placed at the center of the Y-maze and allowed to roam freely and uninterrupted for 15 min. Behavior was recorded using an ELP USB camera with 2.8–12 mm VARIFOCAL lens (Cat# ELP-USBFHD04H-FV) and analyzed with a custom-written program in Bonsai (Lopes et al., 2015). Number of entries and percentage of alternation were recorded as previously described (Gue et al., 2004).

#### Whisker-Dependent Texture Discrimination

Whisker-dependent texture discrimination test was performed as previously described (Chen et al., 2018). All behavior was recorded using a Basler acA1300-60gm GigE camera and EthoVision XT V10.0. Mice exhibiting insufficient interest (<16 total interactions or <10 s total interaction time) or a column bias (>60% interactions with one column) during encoding were excluded.

#### Prepulse Inhibition of the Acoustic Startle Response

The Kinder Scientific Acoustic Startle system (Cat# SM1000-II) was used to evaluate ASR and PPI. The mouse was acclimated to the restrainer with 65 dB white noise (15 min/days for 2 days). On day 3, it underwent the ASR test, starting with a 5-min acclimation. Then it was exposed to a series of white noise stimuli (70, 80, 90, 100, 110, or 120 dB; 40 ms each, 10 stimuli per loudness) in a pseudo-random order, with a variable inter-trial interval of 10–25 s. A group of five consecutive stimuli (40 ms 120 dB white noise) were presented immediately before and after the 60 trials. Maximum responses were recorded for each trial and averaged for each loudness level.

On day 4, the mouse underwent the PPI test starting with a 5-min acclimation. Then it was exposed to a series of white noise stimuli (seven types, 10 stimuli each) presented in a pseudo-random order, with a variable inter-trial interval of 10–25 s. One of the seven types consisted of a single 40 ms 120 dB white noise (startling stimulus). The remaining six types each consisted of a non-startling prepulse (40 ms white noise, 67, 69, 71, 73, 75, or 77 dB) followed by a 40 ms 120 dB white noise stimulus 100 ms later. A group of five consecutive stimuli (40 ms 120 dB white noise) were presented immediately before and after the 70 trials. Maximum responses were recorded for each trial and averaged for each stimulus type (*R*_max_). Percent inhibition was calculated as $\frac{R_{\max|120\,\text{dB}}-R_{\max|\text{PP}}}{R_{\max|120\,\text{dB}}}\times 100.$

### Mesoscopic *in vivo* Ca^2+^ Imaging

The Snap25-GCaMP6s mouse (P21) was anesthetized with 2.5% isoflurane and placed on a heating pad to maintain body temperature. Ophthalmic ointment was applied to the eyes and 1% lidocaine was applied to the scalp. The scalp was then carefully excised to expose the skull. Cyanoacrylate glue was used to secure two head bars to the skull, one across the back of the skull and the other on the lateral parietal bone. While still anesthetized, the mouse was transferred to a rotating disk apparatus with its head bar fastened to the head fixation platform and allowed to recover for 1 h, after which mesoscopic imaging started. Images were taken through a pair of photographic lenses in tandem (focal length 50 mm, *F* = 1.2 and 5.6, respectively) coupled to a scientific cMOS camera (PCO Edge 5.5, ∼ 6.5 μm pixel resolution; PCO AG, Kelheim, Germany). A pair of blue light-emitting diodes (470 nm, Cat# M470L3, Thorlabs) provide the excitation light, each beam passing through a 480/30 nm bandpass filter (Cat# AT480/30x, Chroma Technology). Emitted fluorescence was collected through a 520/36 nm bandpass filter (Cat# 67-044, Edmund Optics). In each imaging session, 16-bit images (2,560 × 2,160 pixels) were collected at 10 Hz frame rate for 10 min. Each mouse underwent two imaging sessions before being euthanized.

For image analysis, we first used a mask generated from a single image frame to select pixels belonging to the brain for further processing. We computed ΔF/F_0_ per pixel using its mean value throughout the entire time series as F_0_. Then we applied an independent component analysis (ICA) pipeline custom-written in Python to decompose the Ca^2+^ image series into eigenvectors, and manually removed eigenvectors corresponding to vascular and other artifacts. From images re-built with neural eigenvectors we defined domain maps and aligned them to the Allen Brain Atlas to determine to which anatomical regions they belong.

Wavelet coherence between domains was computed using custom-written code based on the software provided by Torrence and Compo^1^. We implemented a morlet wavelet (ω = 4) and functions of wavelet coherence (Grinsted et al., 2004). We computed the wavelet coherence between each pair of domains and used a threshold of 0.4 to define whether they were coherent. In each anatomical region, we defined its coherence index as the percentage of distinct pairs of domains therein that were coherent. Analogously, for each pair of anatomical regions, we defined their mutual coherence index as the percentage of distinct pairs of domains (one from each region) that were coherent. Ca^2+^ events were detected by projecting through the periods of the wavelet space; each event was defined as a significant wavelet structure above the red noise threshold (Torrence and Compo, 1998). Descriptive statistics of each event were then computed, and the median of each feature was reported as a measure of each domain’s behavior.

### Immunohistochemistry for Parvalbumin and Perineuronal Net

Mice were transcardially perfused with 4% paraformaldehyde (PFA) in 0.1 M PBS. Brains were post-fixed in 4% PFA at 4°C overnight and then cryopreserved (30% sucrose, 0.01M PBS, 0.05% NaN_3_). 50 μm-thick coronal sections were cut using a vibratome (Cat# VT1000S, Leica Biosystems), incubated first in a blocking solution (5% normal goat serum, 5% BSA, 0.2% Triton X-100 in PBST), then with a mouse anti-PV primary antibody (MAB1572, Millipore; 1:1000) for 72 h at 4°C, and next with a goat-anti-mouse secondary antibody conjugated to Alexa Fluor 594 (Cat# A-11032, Thermo Fisher Scientific, 1:500) for 2 h at room temperature (RT). Sections were then incubated with biotinylated *Wisteria Floribunda* Lectin (WFL; Cat# B-1355, Vector Laboratories; 1:200) and subsequently with streptavidin conjugated to Alexa Fluor 488 (Cat# S32354, Thermo Fisher Scientific; 1:200) for 2 h at RT. Sections were counterstained with DAPI (1:36,000) and mounted with Vectashield (Cat# H-1000, Vector Laboratories). Images of the barrel cortex were captured on a Zeiss Axio Imager Z2 widefield microscope with apotome using a 10x/0.45 NA objective lens. Cells that were PV+ or surrounded by WFL immunostaining were marked using Stereo Investigator 11 (MBF Bioscience), and their densities were calculated using Neurolucida Explorer 11 (MBF Bioscience).

### Immunohistochemistry for Microglia Density and Morphological Analysis

To examine microglia density, 50 μm-thick coronal sections were incubated first in a peroxidase solution (0.6% H_2_O_2_, 1% methanol, 0.01M PBS), followed by a blocking solution (10% normal donkey serum, 5% BSA, 0.7% PBST), and then with a goat anti-Iba1 primary antibody (Cat# ab5076, Abcam, 1:1000) for 72 h at 4°C. Sections were finally incubated with a biotinylated donkey-anti-goat secondary antibody (Cat# 017-000-121, Jackson ImmunoResearch Laboratories; 1:200) for 2 h at RT, and treated with the VECTASTAIN Elite ABC system (Cat# PK-6100, Vector Laboratories). Sections were then counterstained with DAPI (1:36,000) and mounted onto slides with Fluoromount-G (Cat# 00-4958-02, Thermo Fisher Scientific). Images were captured on a Zeiss Axio Imager Z2 widefield microscope using a 10x/0.45 NA objective. Microglia density was quantified using Neurolucida Explorer 11 (MBF Bioscience).

To examine microglia morphology, 50 μm-thick coronal sections were incubated first in the blocking solution and then with a goat anti-Iba1 primary antibody as above. Sections were then incubated with a donkey-anti-goat secondary antibody conjugated to Alexa Fluor 488 (Cat# A11055, Thermo Fisher Scientific, 1:1000), counterstained and mounted as above. Images were taken on a Zeiss 880 confocal microscope using a 63x/1.4 NA oil-immersion objective and analyzed using IMARIS (Bitplane). Microglia with all processes entirely contained within the image stack were identified, and a ROI was generated around each using the IMARIS surface function. Microglia were reconstructed with the IMARIS filament tracer function using autopath (no loop allowed), with spurious or missing filament traces manually removed or appended. The number of terminal points, total process length, convex hull volume, and number of Sholl intersections were exported from IMARIS.

### *In vivo* Transcranial Imaging of Dendritic Spine and Microglial Dynamics

The procedure for thin skull preparation, imaging, spine and microglia analyses were performed as described previously (Davalos et al., 2005; Xu et al., 2009). Imaging was performed on a Bruker Ultima Investigator 2P microscope equipped with a Spectra-Physics Mai Tai laser operating at 940 nm. Spine image stacks were acquired using a 16x/0.8 NA water immersion objective (CFI75 LWD 16X W, Nikon Instruments) at 12x optical zoom with z-step = 1 μm and analyzed using ImageJ. For microglial imaging, a time series of a single image plane was acquired with a 40x/0.8 NA lens at 4x optical zoom at an interval of 10 s for 25 min and analyzed using IMARIS. Typically, 7–11 terminal tips were analyzed per cell. Terminal tip length was calculated by measuring the distance from the tip to the first node using the IMARIS semi-automatic tracer function every 5 min. Net length change was calculated by summing the difference in length between subsequent timepoints for each terminal tip across 25 min. Absolute length change was calculated by summing the absolute value of the difference in length change between subsequent time points for each terminal tip across 25 min.

### Statistical Analyses

Statistical analyses were performed using GraphPad Prism 8.4. The Shapiro–Wilk test was used to check for normality and to determine whether parametric or non-parametric tests were to be used. Data presented as bar graphs show the mean with error bars or shades representing the SEM; data presented as boxplots show the minimum, the first quartile, the median, the third quartile, and the maximum.

## Results

### Perinatal Penicillin Exposure Impairs Texture Discrimination in Adolescent Mice

**FIGURE 1:**
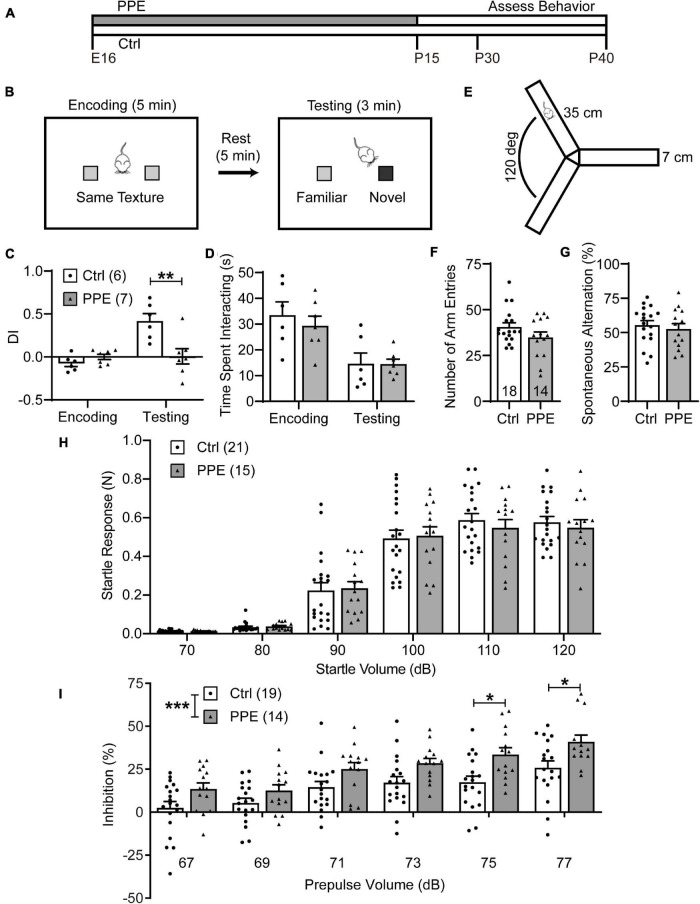
PPE impairs WTD and sensorimotor gating, but not exploratory activity or working memory. **(A)** Timeline of PPE and behavioral assessments. **(B)** Schematic of WTD task. **(C)** PPE mice behave similarly as controls (Ctrl) in the encoding phase [unpaired *t*-test, *t*(11) = 1.549, *p* = 0.150], but show significantly diminished novelty-preference in the testing phase [unpaired *t*-test, *t*(11) = 3.279, *p* < 0.01]. **(D)** Ctrl and PPE mice spend a comparable amount of time investigating the textures in both the encoding [unpaired *t*-test, *t*(11) = 0.6563, *p* = 0.525] and the testing phase [unpaired *t*-test, *t*(11) = 0.0295, *p* = 0.977]. **(E)** Schematic of the Y-maze arena. **(F)** PPE does not affect the number of arm entries (Mann–Whitney test, *U* = 96, *p* = 0.262). **(G)** PPE does not affect the percentage of spontaneous alternation [unpaired *t*-test, *t*(30) = 0.5636, *p* = 0.577]. **(H)** Louder acoustic stimulus evokes greater ASR in both Ctrl and PPE mice [two-way ANOVA, main effect of volume, *F*(5,204) = 127.4, *p* < 1 × 10^–4^], with no significant difference between the two groups [two-way ANOVA, main effect of treatment, *F*(1,204) = 0.1207, *p* = 0.729]. **(I)** Louder prepulse stimulus attenuates ASR more in both Ctrl and PPE mice [two-way ANOVA, main effect of prepulse volume, *F*(5,186) = 15.09, *p* < 1 × 10^–4^]. PPE mice exhibit significantly greater inhibition of ASR than Ctrl mice [two-way ANOVA, main effect of treatment, *F*(1,186) = 32.85, *p* < 1 × 10^–4^]. *Post hoc* Bonferroni’s multiple comparison test shows that with 75 and 77 dB prepulses, the mean inhibition is significantly different between PPE and Ctrl mice (*p* < 0.05 for both). Hereinafter unless stated otherwise, *n* = number of mice; *p < 0.05, **p < 0.01, ***p < 0.001 or less.

Mice use their whiskers, highly specialized and sensitive peripheral tactile sensing structures, to acquire tactile and spatial information about their surroundings. This information is relayed to the barrel cortex (S1BF) for further processing, which ultimately shapes behavioral responses (Petersen, 2007; Feldmeyer et al., 2013). To examine how PPE affects sensory processing, we gave penicillin (31 mg/kg bodyweight per day) to pregnant dams in their drinking water starting on embryonic day (E) 16 to postnatal day (P) 15 (Figure 1A). As penicillin is absorbed by mammalian cells, crosses the placenta, and is secreted in milk, pups were exposed to penicillin both *in utero* and via nursing (Chung et al., 2002; Doi and Chambers, 2015). This delivery method does not affect the daily liquid intake of the dam (Supplementary Figure 1) and avoids the stress of gavage or injection to the pups.

PPE mice grossly followed the same developmental trajectory as controls (Supplementary Figure 2). When PPE mice reached adolescence (1 month of age), we subjected them to the whisker-dependent texture discrimination (WTD) task (Figure 1B), a behavioral paradigm that takes advantage of the rodent’s innate preference for novelty to assay sensory processing (Wu et al., 2013; Chen et al., 2018). While control mice spent significantly more time investigating the column coated with the novel texture than the one with the familiar texture, PPE mice lost such preference (Supplementary Figure 3). To quantify such preference, we defined a discrimination index (DI) as the amount of time spent interacting with one texture (e.g., the novel) minus that with the other texture (e.g., the familiar), normalized by the total amount of interaction time. PPE mice exhibited comparable DI in the encoding phase as controls, but significantly lower DI in the testing phase than controls (Figure 1C). The diminution of DI in PPE mice is not due to a lack of interest in texture exploration, as they spent a comparable time investigating the columns as controls during both encoding and testing (Figure 1D). Nor is the lack of novelty preference due to increased anxiety or impaired working memory, as PPE mice performed normally in the open field maze (Supplementary Figure 4) and the Y-maze spontaneous alternation test (Figures 1E–G).

### Perinatal Penicillin Exposure Alters Sensorimotor Gating in Adolescent Mice

Sensorimotor gating refers to the phenomenon whereby sensory stimuli suppress motor responses. It can be assayed via the prepulse inhibition (PPI) of the acoustic startle response (ASR), in which a non-startling acoustic stimulus (prepulse) preceding a startling stimulus attenuates the startle response to the latter (Ouagazzal and Meziane, 2012). We found that higher startle volumes elicited increasingly larger ASRs in both control and PPE mice, without significant difference in the mean startle responses between the two groups (Figure 1H). In the PPI test, we observed that increased prepulse volumes elicited significantly greater percent inhibition of ASR in both control and PPE animals. Interestingly, PPE mice exhibited significantly higher percent inhibition than controls. The difference in percent inhibition was most significant when the 120-dB startle stimulus was preceded by the loudest prepulse stimuli (Figure 1I).

### Perinatal Penicillin Exposure Alters the Functional Network of Cortical Neurons in Adolescent Mice

**FIGURE 2:**
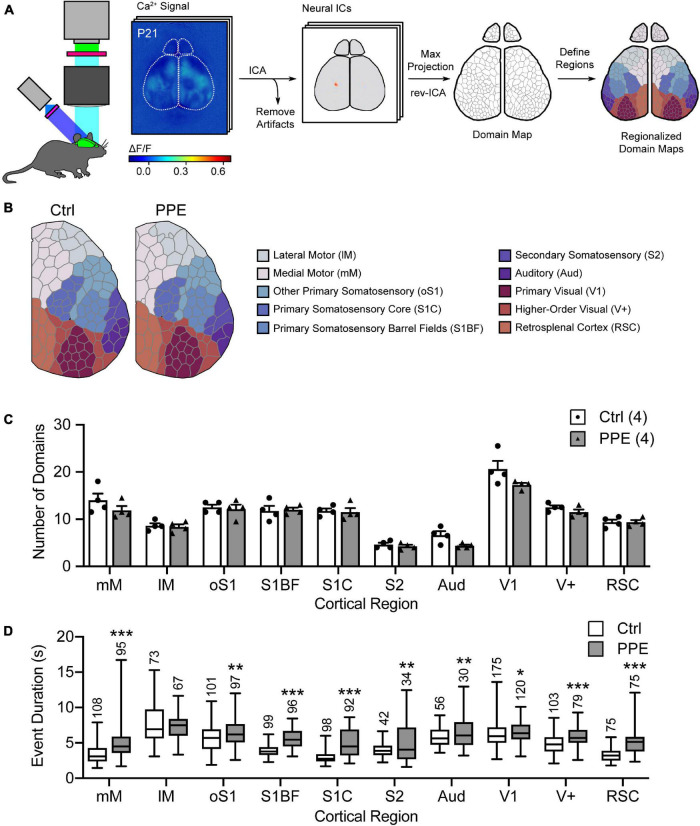
PPE affects mesoscopic spatiotemporal organizations of cortical neuronal activities. **(A)** Schematic of the mesoscale Ca^2+^ imaging and data processing pipeline. **(B)** Representative regionalized domain maps of Ctrl and PPE mouse cortices. **(C)** PPE does not change the number of domains per cortical region (df = 6, *q* > 0.4 for all regions). **(D)** PPE significantly increases Ca^2+^ event duration in domains of S1BF, S1C, RSC, mM (*q* < 1 × 10^–6^), V+ (*q* < 1 × 10^–5^), oS1, S2, Aud (*q* < 0.01), and V1 (*q* < 0.05); df = 1695 for all comparisons. All *q*-values are given by multiple *t*-tests with FDR correction using the two-stage Benjamini-Krieger-Yekutieli procedure. *n* = number of mice **(C)** or number of domains **(D)**. 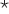
*p* < 0.05, 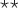
*p* < 0.01, 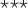
*p* < 0.001 or less.

Next, we used mesoscopic *in vivo* Ca^2+^ imaging (Vanni and Murphy, 2014) to monitor cortical neural activities in early adolescent (P21) control and PPE Snap25-GCaMP6s mice. These mice express the genetically encoded Ca^2+^ indicator GCaMP6s across cortical neurons. With an intact skull, the illumination intensity is greatest at the superficial layers. Furthermore, due to light scattering in the brain tissue, signals from neuronal somata in deep layers are widespread at the surface (Waters, 2020). Therefore, neurons in superficial layers, as well as apical dendrites of deeper layer neurons, together contribute most significantly to the recorded signal. We used an independent component analysis pipeline (pySEAS) (Weiser et al., 2021) to isolate neuronal activities and identify distinct functional domains. Domain maps generated by pySEAS have been shown to be very similar across imaging sessions within the same animal, and share structural similarities across animals (Weiser et al., 2021). We further assigned each functional domain to one of the ten cortical regions defined by the Allen Brain Atlas (Figure 2A). We found that PPE and control mice had comparable number of domains per cortical region (Figures 2B,C). Using coherence analysis (Grinsted et al., 2004) to quantify synchronous activities, we further showed that PPE did not significantly alter the level of coherence either between domains within a cortical region or between pairs of cortical regions (Supplementary Figure 5). However, PPE increased the duration of spontaneous Ca^2+^ events in most cortical regions including the barrel cortex, which we further investigated below (Figure 2D).

### Perinatal Penicillin Exposure Perturbs the Maturation of Parvalbumin-Expressing Inhibitory Interneurons in the Barrel Cortex

**FIGURE 3:**
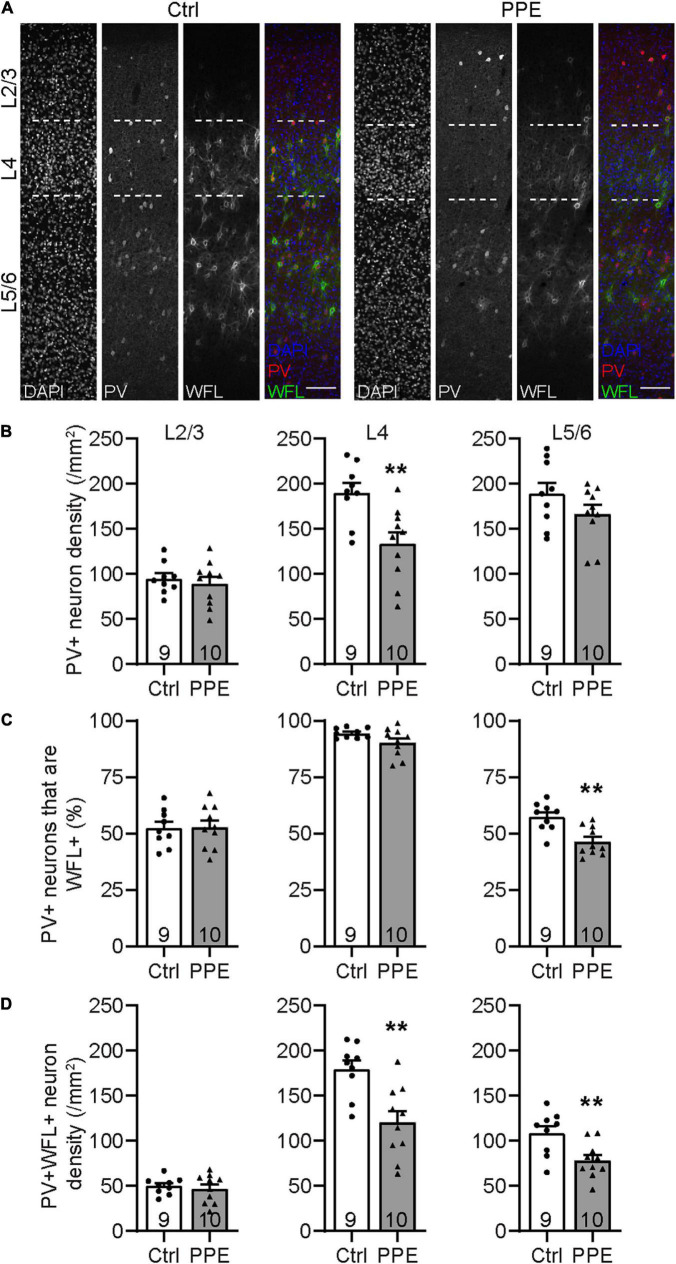
PPE perturbs the maturation of S1BF PV+ INs during adolescence. **(A)** Representative images of immunohistochemistry for PV and WFL in the S1BF of Ctrl and PPE mice. Scale bar = 100 μm. **(B)** PPE significantly decreases PV+ cell density in L4 [*t*(17) = 3.304, *p* < 0.01], but not L2/3 [*t*(17) = 0.6082, *p* = 0.551] or L5/6 [*t*(17) = 1.448, *p* = 0.166]. Unpaired *t*-tests for all. **(C)** PPE significantly decreases the percentage of PV+ cells that are WFL+ in L5/6 [unpaired *t*-test, *t*(17) = 3.735, *p* < 0.01], but not L2/3 [unpaired *t*-test, *t*(17) = 0.0883, *p* = 0.931] or L4 [unpaired *t*-test with Welch’s correction, *t*(11.3) = 1.974, *p* = 0.073]. **(D)** PPE significantly decreases the density of neurons that are both PV+ and WFL+ in L4 [*t*(17) = 3.645, *p* < 0.01] and L5/6 [*t*(17) = 3.005, *p* < 0.01], but not in L2/3 [*t*(17) = 0.5625, *p* = 0.581]. Unpaired *t*-tests for all. 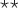
*p* < 0.01.

Parvalbumin-expressing inhibitory interneurons are the predominant type of interneurons in the mammalian cortex (Rudy et al., 2011). They mature postnatally and play important roles in feedforward inhibition and sensory processing (Atallah et al., 2012; Lee et al., 2012). During early adolescent development, perineuronal nets (PNNs; extracellular proteoglycan matrices) assemble around these cells. PNNs are thought to be involved in the closure of critical periods and thus may serve as markers for the functional maturity of cortical circuits (Carulli and Verhaagen, 2021). To determine how PPE affects the development of PV+ INs in the S1BF, we stained brain slices collected from adolescent (P45) mice with an anti-PV antibody and *Wisteria floribunda* lectin/agglutinin (WFL/WFA), a lectin that binds the aggrecan component of PNNs (Figure 3A). We found that the PV+ IN density in S1BF L4, but not L2/3 or L5/6, was significantly lower in PPE mice than in controls (Figure 3B). Additionally, the percentage of PV+ INs that were surrounded by WFL+ PNNs was significantly lower in L5/6, but not L2/3 or L4 of the S1BF of PPE mice compared to controls (Figure 3C). Together, these defects resulted in a significant decrease in the density of S1BF PV+ INs that were surrounded by WFA+ PNNs in deeper cortical layers (L4 and L5/6) in PPE mice as compared with controls (Figure 3D).

### Perinatal Penicillin Exposure Leads to Dendritic Spine Loss in the Barrel Cortex of Adolescent Mice

**FIGURE 4:**
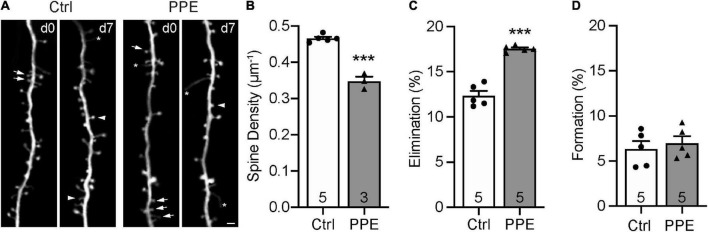
PPE accelerates the pruning of dendritic spines on L5 pyramidal neurons in S1BF during adolescence. **(A)** Examples of the same dendritic segment imaged over 7 days *in vivo* in Ctrl and PPE mice. Arrow: eliminated spine; arrowhead: new spine; asterisk: filopodium. Scale bar: 2 μm. **(B)** PPE significantly decreases spine density [unpaired *t*-test, *t*(6) = 10.77, *p* < 1 × 10^–4^]. **(C)** PPE significantly increases spine elimination over 7 days [unpaired *t*-test with Welch’s correction, *t*(4.639) = 9.363, *p* < 0.001]. **(D)** PPE does not affect spine formation [unpaired *t*-test, *t*(8) = 0.5474, *p* = 0.599]. 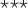
*p* < 0.001 or less.

Dendritic spines are highly specialized membranous structures that protrude out of the dendritic branches. Abnormal spine density and morphology are observed in a number of neuropsychiatric disorders (Blanpied and Ehlers, 2004; Penzes et al., 2011). To investigate how PPE affects dendritic spines in the S1BF, exposed *Thy1*-YFP-H line (Feng et al., 2000) mouse pups to PPE. These mice expressed cytoplasmic yellow fluorescent protein (YFP) in a sparse subset of L5 pyramidal neurons in the cortex, which enabled us to image dendritic spines *in vivo* using transcranial two-photon microscopy. We imaged spines on apical dendrites (within 150 μm below the pial surface) in S1BF of control and PPE mice at 1 month of age (Figure 4A) and found that the spine density in control mice was significantly higher than that in PPE mice (Figure 4B). Following the same dendrites over time, we further showed that spine elimination over 7 days was significantly elevated in PPE mice compared to controls (Figure 4C). In contrast, spine formation over the same period was not affected by PPE (Figure 4D).

### Perinatal Penicillin Exposure Increases the Ramification and Territorial Overlap of Microglia in the Barrel Cortex of Adolescent Mice

**FIGURE 5:**
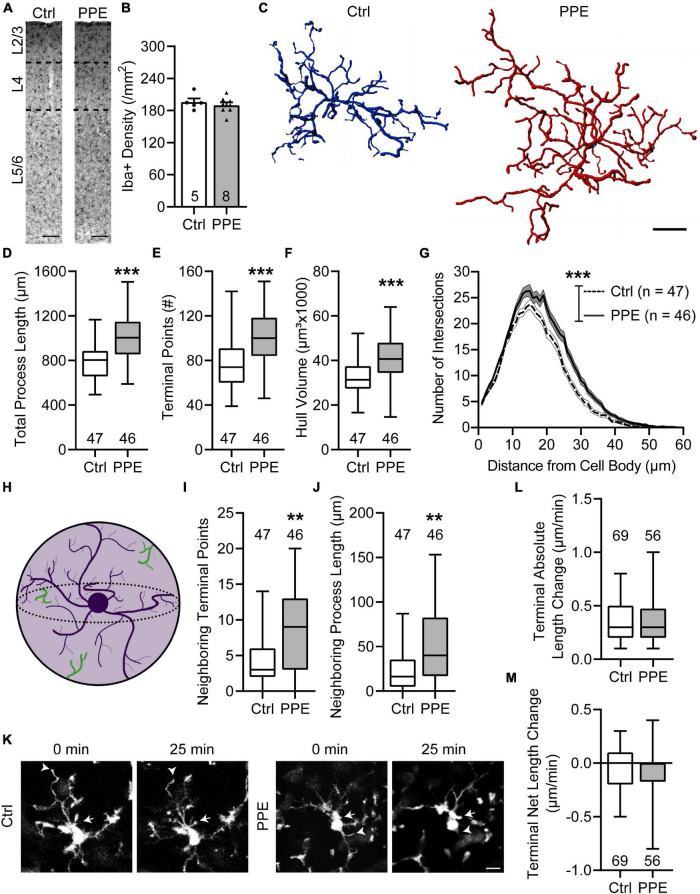
PPE increases the ramification and territorial coverage of microglia in adolescent S1BF. **(A)** Representative images of immunolabeling for Iba1 in Ctrl and PPE mice. Scale bar = 100 μm. **(B)** Microglia density is comparable between Ctrl and PPE mice [unpaired *t*-test, *t*(11) = 0.7046, *p* = 0.496]. **(C)** Representative three-dimensional reconstructions of Ctrl and PPE microglia. Scale bar = 10 μm. **(D–F)** PPE significantly increases the total process length [unpaired *t*-test with Welch’s correction, *t*(82.77) = 4.861, *p* < 1 × 10^–4^; **(D)**], the number of terminal points [unpaired *t*-test, *t*(91) = 4.634, *p* < 1 × 10^–4^; **(E)**], and the convex hull volume [unpaired *t*-test, *t*(91) = 3.728, *p* < 0.001; **(F)**]. **(G)** PPE significantly increases microglial ramifications as shown by Sholl analysis [two-way ANOVA, main effect of treatment, *F*(1,5520) = 260.3, *p* < 1 × 10^–4^]. The increase is particularly significant at distances of 13–31 μm from the soma (*p* < 0.05 for all but 15, 16, and 18 μm, *post hoc* Bonferroni multiple comparisons test). **(H)** A cartoon illustrating neighboring microglial processes (green) entering the convex hull (light purple) of one microglia (dark purple). **(I,J)** PPE increases the number of terminal points [Mann–Whitney test, *U* = 692, *p* < 0.01; **(I)**] and the total process length [Mann–Whitney test, *U* = 665, *p* < 0.01; **(J)**] of neighboring microglia within the convex hulls of the traced microglia. **(K)** Example time-lapse *in vivo* 2P images of microglia in Ctrl and PPE mice. Arrows: process extension; arrowheads: process retraction. Scale bar = 10 μm. **(L,M)** PPE does not affect the absolute length change [Mann–Whitney test, *U* = 1826, *p* = 0.592; **(L)**] or the net length change [Mann–Whitney test, *U* = 1841, *p* = 0.645; **(M)**] of microglia terminal processes. *n* = number of mice **(B)**, microglia **(D–J)**, or terminal processes **(L,M)**. 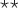
*p* < 0.01, 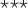
*p* < 0.001 or less.

To evaluate the effect of PPE on microglia, we stained them with an antibody against Iba1, a constitutively expressed microglia marker (Imai and Kohsaka, 2002), in brain slices from adolescent control and PPE mice, and analyzed their density and morphology in S1BF (Figure 5A). We found that PPE affected neither the overall microglia density (Figure 5B) nor any of the layer-specific densities (Supplementary Figure 6). Interestingly, microglia in PPE mice appeared much larger (Figure 5C): they had significantly increased total process lengths (Figure 5D), more termini (Figure 5E), and larger convex hull volume (Figure 5F). Sholl analysis further revealed that microglia in PPE mice had more ramifications than controls, particularly at distances of 13–31 μm from the cell body (Figure 5G). As PPE increased the size of S1BF microglia without affecting their density, we sought to determine whether PPE affected the degree to which microglia respected the parenchymal territories of their neighbors (Figure 5H). Within the convex hulls of traced microglia, we observed significantly more termini (Figure 5I) as well as longer processes belonging to neighboring microglia (Figure 5J).

To investigate how PPE affects the process dynamics of microglia, we performed *in vivo* two-photon imaging of adolescent CX3CR1^GFP/+^ mice with and without PPE (Figure 5K). We found that the tips of microglia were dynamic, with comparable extensions and retractions over 25 min between control and PPE mice (Figure 5L). There was no significant difference in the net change of microglial tip length between control and PPE mice (Figure 5M). Coupled with the significant increase in the number of microglia process termini in PPE mice, these data suggest that the overall dynamics of PPE microglia at the cellular level are significantly increased.

### Perinatal Penicillin Exposure-Induced Behavioral Abnormalities Persist Into Adulthood

**FIGURE 6:**
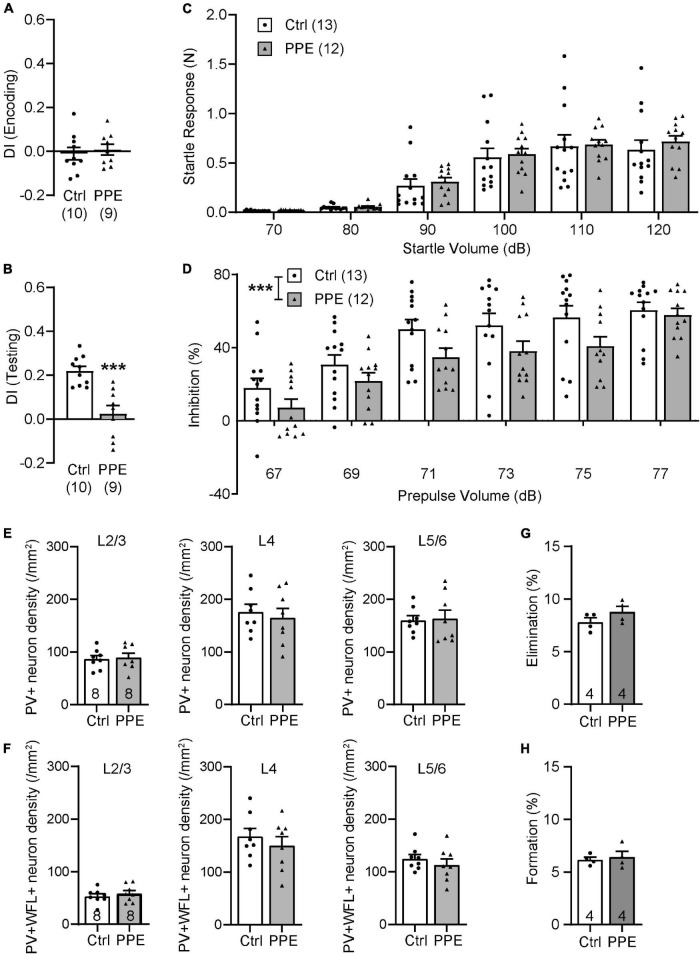
PPE-induced behavioral and neuronal defects persist into adulthood. **(A,B)** Adult PPE mice show no difference in column preference during the encoding phase [**(A)**; unpaired *t*-test, *t*(17) = 0.4792, *p* = 0.638], but significantly diminished novelty-preference in the testing phase [**(B)**; unpaired *t*-test, *t*(17) = 4.647, *p* < 0.001]. **(C)** Louder acoustic stimulus evokes greater ASR in both adult Ctrl and PPE mice [two-way ANOVA, main effect of volume, *F*(5,138) = 43.95, *p* < 1 × 10^–4^], with no significant difference between the two treatment groups [two-way ANOVA, main effect of treatment, *F*(1,138) = 0.6748, *p* = 0.413]. **(D)** Louder prepulse stimulus attenuates ASR more in both adult Ctrl and PPE mice [two-way ANOVA, main effect of prepulse volume, *F*(5,138) = 20.70, *p* < 1 × 10^–4^]. However, adult PPE mice exhibit significantly lower inhibition of ASR than Ctrl mice [two-way ANOVA, main effect of treatment, *F*(1,138) = 13.79, *p* < 0.001]. **(E)** Adult PPE mice have normal density of PV+ neurons in L2/3 [*t*(14) = 0.2776, *p* = 0.785], L4 [*t*(14) = 0.4878, *p* = 0.633], and L5/6 [*t*(14) = 0.1750, *p* = 0.864] of S1BF. Unpaired *t*-tests for all. **(F)** Adult PPE mice have normal density of neurons that are both PV+ and PNN+ in L2/3 [*t*(14) = 0.6907, *p* = 0.501], L4 [*t*(14) = 0.7806, *p* = 0.448], and L5/6 [*t*(14) = 0.8416, *p* = 0.414] of S1BF. Unpaired *t*-tests for all. **(G,H)** PPE does not significantly affect spine formation [*t*(6) = 0.4189, *p* = 0.690] or elimination [*t*(6) = 1.452, *p* = 0.197] over 7 days in adult mice. Unpaired *t*-test for both. 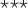
*p* < 0.001 or less.

To determine whether the effects of PPE are transient or long-lasting, we examined behavioral and neuronal phenotypes in adult mice (P90). We found that adult PPE mice continued to exhibit behavioral defects in sensory processing and sensorimotor gating. Adult PPE mice behaved normally during the encoding phase of the WTD task (Figure 6A), but lost the preference for the novel texture in the discrimination phase (Figure 6B). Interestingly, while adult PPE mice exhibited comparable ASR as age-matched controls (Figure 6C), they had lower PPI (Figure 6D), in contrast to adolescent PPE mice (Figure 1I). However, neither the density of PV+ INs nor the density of PV+ INs surrounded by WFL+ PNNs in all layers of S1BF was significantly different between adult PPE mice and controls (Figures 6E,F). Finally, adult PPE mice showed comparable dendritic spine dynamics with aged-matched controls (Figures 6G,H). Together, these results suggest that, while PPE-induced deficits in PV+ IN maturation and in cortical dendritic spine dynamics were transient, the behavioral impairment persists beyond adolescence into adulthood.

## Discussion

In humans, late embryonic development and early postnatal life (especially the first year) is marked by pronounced structural and functional changes in the brain, including synaptogenesis, neural circuit maturation, as well as the maturation of microglia. It is also within this period that epidemiological work has revealed significant correlations between antibiotic exposure and lasting neurocognitive issues (Atladottir et al., 2012; Slykerman et al., 2017). In the mouse, an analogous neurodevelopmental period, comprising much of these processes, occurs in the final days of gestation and first few weeks of life (Stagni et al., 2015). In this study, we adopted an antibiotic exposure regimen that had been reported to elicit penicillin-associated defects in social behaviors, localized increases in neural transcription of proinflammatory cytokines, and perturbations to microbiota communities in mice (Leclercq et al., 2017). The dosage (31 mg/kg per day) is comparable with the usual therapeutic dosage for children (25–50 mg/kg per day, divided into three doses) and for adults (125–500 mg, four times per day) (Doi and Chambers, 2015). Various previous studies on perinatal antibiotic exposure used slightly longer time windows ending at P21 or P28 (Cox et al., 2014; Leclercq et al., 2017; Miyoshi et al., 2017; Lynn et al., 2018). We ended penicillin exposure at P15 to better align with the aforementioned neurodevelopmental period; this choice also avoids the complication that older pups may drink from the water bottle and thus be exposed to penicillin directly. We found penicillin-associated behavioral defects in sensory processing as well as neurobiological defects in the somatosensory cortex. Notably, some previous studies also restricted antibiotic exposure to the prenatal period or adopted a shorter postnatal exposure time window, and still found altered behavioral or immune responses in the offspring (Russell et al., 2013; Tochitani et al., 2016; Lynn et al., 2018; O’Connor et al., 2021). Whether different regimens differentially affect neuronal maturation, synaptic dynamics, and neuronal activities in the offspring remain to be investigated.

We found in adolescent PPE mice increased PPI of the ASR, but decreased PPI in adult PPE mice. This result is intriguing. The neural mechanism underlying PPI in rodents is very complex, involving multiple cortical and subcortical regions (Swerdlow et al., 2001). Further studies are necessary to dissect the circuit basis of such disparate changes in adolescent vs. adult PPE mice. We also found that PPE mice had decreased investigative preference for a novel texture in the WTD task. This deficit is accompanied by an increase in the spontaneous Ca signals in multiple cortical regions including S1BF. In a previous work (Lu et al., 2021), we showed that increased spontaneous neuronal activity in S1BF of stressed mice is associated with decreased signal-to-noise ratio (SNR) in stimulus-associated neuronal activities and impaired texture discrimination. A similar reduction in SNR may occur in PPE mice, potentially related to the decreased inhibitory inputs (see below). Furthermore, the similarity in the cytoarchitectonics across cortical regions suggests that other sensory areas may be analogously affected.

The behavioral phenotypes we observed suggest that PPE broadly affects sensory processing. Abnormal sensory processing (including sensory sensitivity and active sensing) may be correlated with, or contribute to, neuropsychiatric disorders including ASD, depression, anxiety, and obsessive-compulsive disorder (Harrison et al., 2019). In particular, abnormal sensory experiences, including those in the tactile domain, are frequent in ASD patients, and somatosensory deficits have been reported in multiple mouse models of ASD (Balasco et al., 2019). In these diseases as well as in our PPE mouse model, it is possible that sensory processing deficit is one of the co-morbid conditions, because the neural circuits associated with sensory processing and those associated with other behaviors (e.g., social behaviors) share the same set of molecular and cellular mechanisms, and genetic or environmental perturbations to such underlying mechanisms cause deficits across different circuits simultaneously. Alternatively, as defective sensory processing prevents the animal from properly perceiving environmental and social cues for appropriate reactions, it may indeed be the direct trigger of other behavioral symptoms. Thus, it has been speculated that sensory processing aberrations are part of the etiology of neuropsychiatric disorders (Harrison et al., 2019). If so, early life penicillin exposure may increase the susceptibility of children to neuropsychiatric disorders, consistent with findings in epidemiological studies.

Inhibitory interneurons are pivotal regulators of neural circuit development and function by modulating the activities of local neurons (Le Magueresse and Monyer, 2013; Tremblay et al., 2016). PV+ INs are the predominant type of INs in the cortex (Rudy et al., 2011). They mature postnatally (del Rio et al., 1994) and are believed to be particularly vulnerable to developmental perturbations; their dysfunction is thought to play a large part in various neuropsychiatric disorders (Marin, 2012; Ferguson and Gao, 2018). The finding of decreased density of PV+ INs surrounded by PNNs in the adolescent mouse S1BF suggests PPE affects the maturation of PV+ INs, which is consistent with Ca^2+^ imaging results showing increased spontaneous activity therein. Furthermore, PV+ INs play important roles in sensory processing: their activation facilitates perception, and their impairment is associated with defective sensory discrimination (Lee et al., 2012; Chen et al., 2018). Thus, decreased PV+ IN maturation may be one of the contributors to sensory processing impairments in adolescent PPE mice. Although the density of PV+ INs surrounded by PNNs in S1BF of PPE mice is normalized by P90, the impaired texture discrimination in these mice suggests that the transient perturbation to PV+ IN maturation disrupts the neural circuit in a permanent way. The exact site and nature of the PPE-induced circuit alterations awaits future studies.

Both synaptic and microglial abnormalities have been found in developmental neurological disorders (Frick et al., 2013; Forrest et al., 2018). Accumulating evidence has shown that microglia are important regulators of synaptic activity, dynamics, and plasticity (Tremblay et al., 2010; Rogers et al., 2011; Parkhurst et al., 2013; Sipe et al., 2016). We found that microglia in the PPE mouse cortex are hyper-ramified with higher process dynamics, and such microglial changes accompany the increases in spine elimination. Earlier studies have shown that microglia facilitate synaptic pruning by complement activation and phagocytosis during postnatal development (Paolicelli et al., 2011; Schafer et al., 2012). Conversely, they listen to and feedback on neuronal activities (Badimon et al., 2020; Umpierre and Wu, 2020). Thus, whether microglia in the PPE brain actively remove dendritic spines or simply respond to altered neuronal activity and synaptic elimination remains to be elucidated.

Human data suggest that penicillins administered to lactating women appear in trace quantities, not in therapeutic concentration, in the milk (Anderson, 1977; Chung et al., 2002). Despite inter-species differences in milk protein and lipid composition, milk pH, drug transporter systems, and anatomy (Anderson, 2018), the amount of penicillin V taken up by mouse pups from the milk is expected to be very low as well. Furthermore, little penicillin V can penetrate into the cerebrospinal fluid (Geddes et al., 2017). Therefore, the observed neural and behavioral effects likely do not result from a direct impact of penicillin V on the pup’s physiology, but rather derive indirectly from penicillin-induced dysbiosis in the dam. As the pup acquires its gut microbiota from the dam, the altered microbiota may affect neural development and behavior in the pup via various gut-brain signaling routes, including direct neural connections by the vagus nerve as well as indirectly through microbial metabolites, peptides produced by gastrointestinal cells, and immune signaling elicited by bacterial antigens (Gars et al., 2021). The exact signaling mechanism underlying PPE-induced neural and behavioral phenotypes remains to be elucidated.

## Data Availability Statement

The raw data supporting the conclusions of this article will be made available by the authors, without undue reservation.

## Ethics Statement

The animal study was reviewed and approved by the Institutional Animal Care and Use Committee (IACUC), University of California, Santa Cruz.

## Author Contributions

YZ, JL, and JP designed the study and wrote the manuscript with input from all other authors. JP, JL, BM, TL, and MT performed the experiments and analyzed the data. BM, SW, and JA developed the mesoscopic imaging and analysis pipeline. All authors contributed to the article and approved the submitted version.

## Conflict of Interest

The authors declare that the research was conducted in the absence of any commercial or financial relationships that could be construed as a potential conflict of interest.

## Publisher’s Note

All claims expressed in this article are solely those of the authors and do not necessarily represent those of their affiliated organizations, or those of the publisher, the editors and the reviewers. Any product that may be evaluated in this article, or claim that may be made by its manufacturer, is not guaranteed or endorsed by the publisher.

## Footnotes

1
http://paos.colorado.edu/research/wavelets/
