# Supplementary material for: Perinatal Penicillin Exposure Affects Cortical Development and Sensory Processing
Source: Front Mol Neurosci. 2021 Dec 22;14:704219. doi: 10.3389/fnmol.2021.704219 (PMC8727458; doi:10.3389/fnmol.2021.704219)
Supplement: Supplementary file 1 [file Image_1.pdf]

Supplementary Material

1 Supplementary Methods

Open field maze (OFM)

The OFM arena is a 44 cm × 44 cm × 44 cm Plexiglas cage covered with a 1 cm-thick layer of woodchip bedding. During the test, the subject mouse was placed at the center of the arena and allowed to roam freely and uninterrupted for 10 min. Behavior was recorded using a Basler acA1300-60gm GigE camera (Cat# 106200) and analyzed with EthoVision XT V10.0 (Noldus). Distance traveled, time spent moving, velocity, time spent in the arena center and periphery were measured.

2 Supplementary Figures

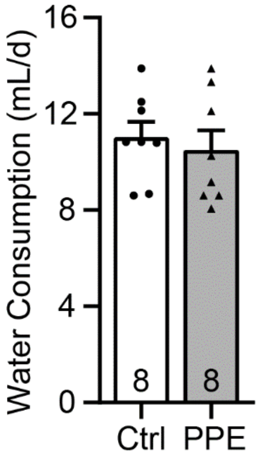

Supplementary Figure 1. PPE does not affect water consumption by pregnant/nursing dams. Over the course of PPE treatment, the average amount of water consumed by pregnant/nursing dams are comparable between Ctrl and PPE groups (unpaired *t*-test, $t(14) = 0.5104$, $p = 0.618$). n = number of mice.

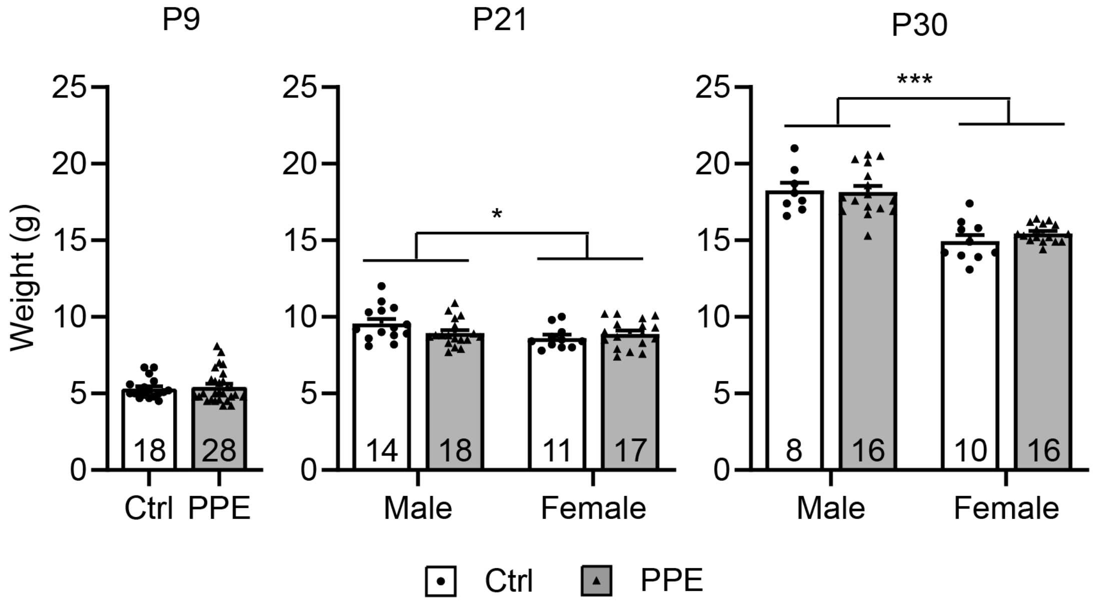

Supplementary Figure 2. PPE does not affect weight across early postnatal development. The weights of PPE and Ctrl pups (both sexes) are comparable at P9 (Mann-Whitney test, $U = 237.5$, $p = 0.750$). At P21 there is no significant difference between treatment conditions (two-way ANOVA, main effect of treatment, $F(1,56) = 0.5081$, $p = 0.479$), but male pups are significantly heavier than females (two-way ANOVA, main effect of sex, $F(1,56) = 4.085$, $p < 0.05$). The same holds for P30 pups (two-way ANOVA, main effect of treatment, $F(1,46) = 0.3113$, $p = 0.580$; main effect of sex, $F(1,46) = 64.77$, $p < 1\times10^{-4}$). n = number of mice.

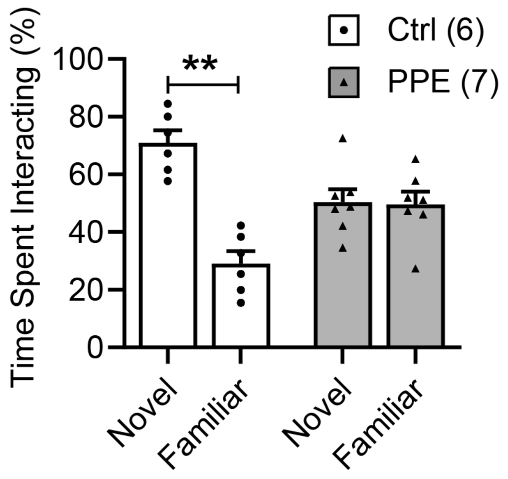

Supplementary Figure 3. PPE suppresses the novelty preference in the WTD task. Ctrl mice spend a significantly higher percentage of time interacting with the novel texture than the familiar one (paired *t*-test, $t(5) = 4.866$, $p < 0.01$), but PPE mice exhibit no such difference (paired *t*-test, $t(6) = 0.093$, $p = 0.929$). n = number of mice.

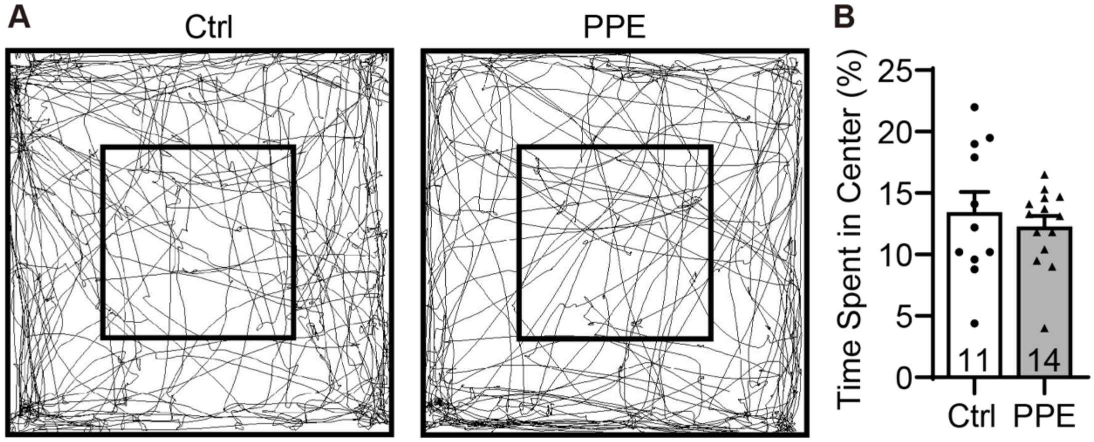

Supplementary Figure 4. PPE does not affect anxiety-like behavior. (A) Example movement trajectories of a Ctrl and a PPE mouse in the OFM arena. **(B)** The percent time spent in the center of the arena does not differ between Ctrl and PPE mice (unpaired *t*-test, $t(23) = 0.6677$, $p = 0.511$). n = number of mice.

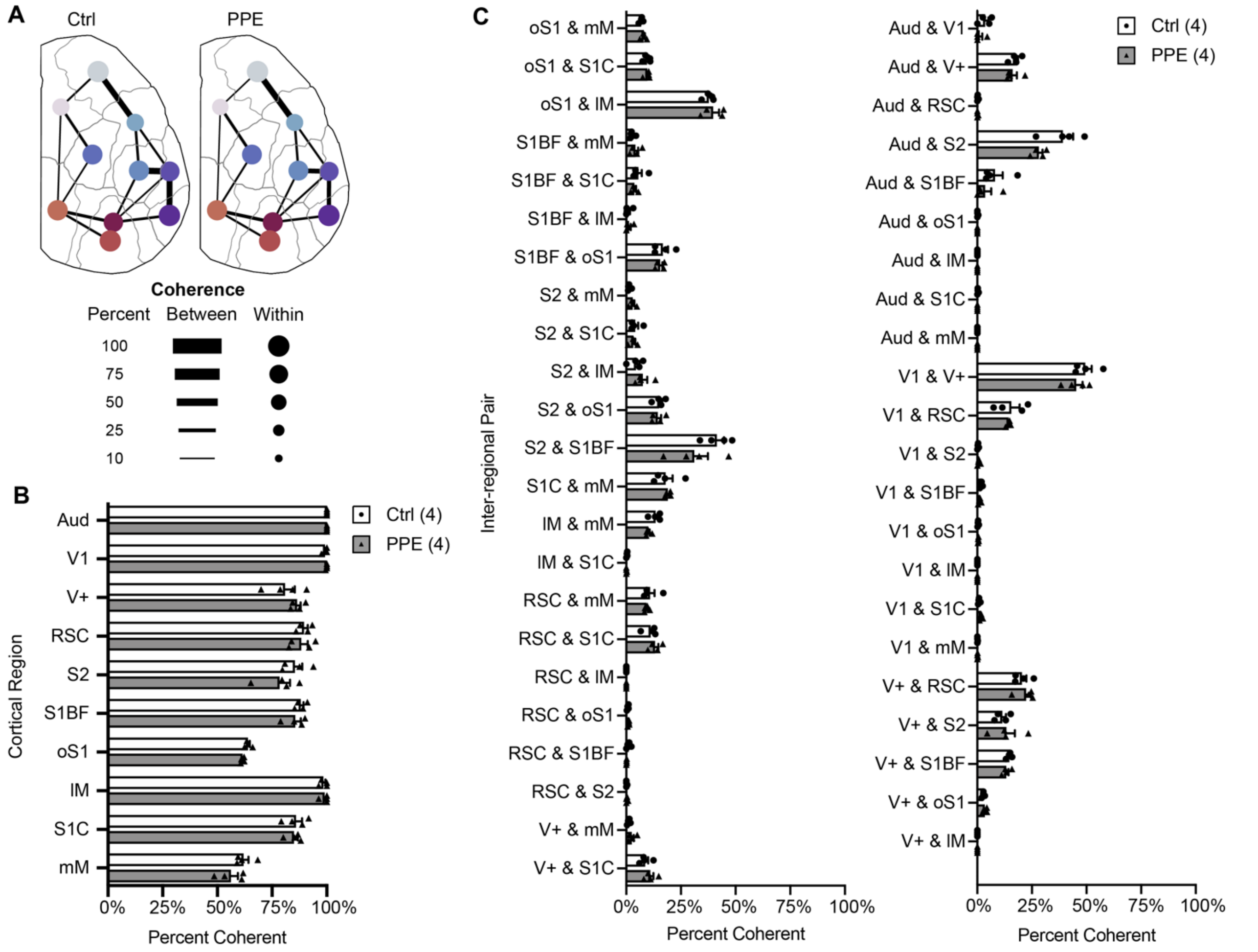

Supplementary Figure 5. PPE does not affect coherence within or between cortical regions. (A) Example of coherence map between cortical regions. **(B)** The coherence level between constituent domains of each cortical region ($q > 0.34$ for all regions). **(C)** The coherence level between pairs of cortical regions ($q > 0.668$ for all regional pairs). All q-values are given by multiple t-tests with FDR correction using the two-stage Benjamini-Krieger-Yekutieli procedure. n = number of mice.

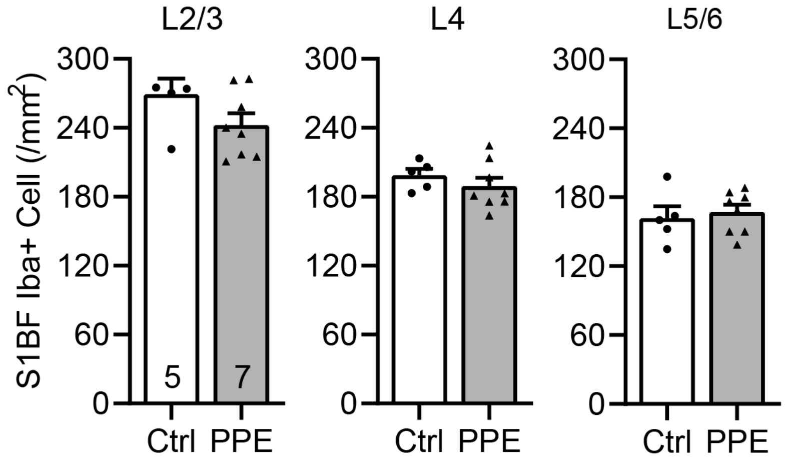

Supplementary Figure 6. PPE does not affect the density of Iba1+ microglia in any layer of the S1BF. The densities of microglia are comparable between Ctrl and PPE mice within L2/3 ($p = 0.137$), L4 ($p = 0.385$), and L5/6 ($p = 0.657$). Unpaired t-test for all. n = number of mice.
